# The Last Days Preceding the Congress

**Published:** 1893-07

**Authors:** 


### THE LAST DAYS PRECEDING THE CONGRESS.

WHEN this number is distributed among our readers the last month preceding that in which the World's Columbian Dental Congress is to convene will have commenced its rapidly-passing weeks.

A wise person will not postpone securing a temporary home at Chicago until the last moment. While the local committee assures us that accommodations are ample and to be procured at reasonable rates, they must be secured in advance. The committee of the Columbia Dental Club have widely advertised the fact that they are prepared to receive applications for rooms. It is hoped that there will be an immediate response to this, and in numbers to warrant the general expectation, long entertained, of the largest dental meeting ever held in this or any other country.

It would seem almost a waste of energy to urge the duty resting upon every dentist to make the sacrifice of money and time to be present. The Congress promises to be the one event in professional life, and with the unequalled attraction of the Exhibition added, it should prove the most powerful educational and social stimulant that dentistry has witnessed or is likely to experience in many generations.

It has been wisely stated in the circular of the Chicago Club that the length of the visit should not be limited to the week of the Congress. The Fair should be largely in the background before that is entered upon. Hence, a week in advance will give time to satisfy, in some degree, the desire to see the Exhibition, and give a zest for the more serious labor of the meeting. Conventions have always suffered when brought in competition with extraordinary attractions elsewhere, and it will be so in Chicago unless this advice be followed.

The work of the various committees has been mainly completed, and the few short weeks yet remaining will be occupied in closing up minor details.

Let each dentist, whether active in scientific work or in the routine of practice, for once close the office during August and make Chicago his abiding-place for the largest portion of that month. The personal sacrifices made, we feel assured, will in the future mark a period in every dentist's personal history, and one he would not willingly have blotted from memory.

